# Immobilization of a biostimulator microbial consortium on bacterial cellulose and its effect on onion growth, soil nutrient status and the microbial community

**DOI:** 10.1007/s11274-025-04739-3

**Published:** 2026-02-23

**Authors:** Rabaa Yaseen

**Affiliations:** Soil Fertility and Microbiology Department, Water Resources and Desert Soils Division, Desert Research Center, Cairo, 11753 Egypt

**Keywords:** Encapsulation, Nanocellulose, Bioinoculant, Allium cepa, Microbiome

## Abstract

The immobilization of plant growth-promoting rhizobacteria (PGPR) in biodegradable polymeric networks is a promising strategy for protecting them from environmental stresses and optimizing their plant-beneficial functions. In this study, a microbial consortium of *Pseudomonas stutzeri* and *Beijerinckia mobilis* was immobilized in bacterial cellulose (BC) produced by *Komagataeibacter xylinus*. Its efficacy was evaluated in onion (*Allium cepa*) under three treatments: uninoculated control, free cells, and immobilized cells, during a field trial conducted in the 2025 winter cropping season at El-Kharga Oasis, New Valley Governorate, Egypt. Both inoculation forms significantly (*P* < 0.05) enhanced plant growth, nutrient uptake, and soil fertility, with immobilized cells increasing bulb yield by 44.9% over the control and outperforming free cells. Treatments elevated N, P, K levels in bulbs and soil, with immobilized cells showing superior nutrient mobilization. The microbial diversity in the onion rhizosphere was assessed at harvest. Ecological diversity indices revealed that bacterial treatments, especially in immobilized form, were associated with a short-term reduction in overall microbial diversity, reflecting selective enrichment of plant-beneficial taxa while suppressing non-beneficial competitors. This functional shift enhanced rhizosphere efficiency without long-term detriment to soil health, as confirmed by post-harvest observations. The novelty of this work lies in the field-scale validation of BC as a biodegradable, highly porous carrier that protects PGPR under arid conditions while simultaneously modulating rhizosphere communities. This study highlights microbial immobilization in BC as a robust, eco-friendly approach to enhance crop yield and soil nutrient dynamics, offering a scalable strategy for sustainable agriculture. Photo 1 Graphical illustration showing PGPR immobilization in biocellulose and its effect on onion growth and nutrient uptake through microbiome modulation.

**Graphical abstract:**

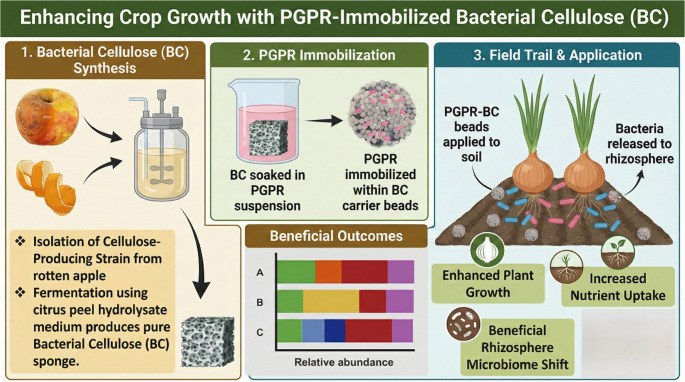

## Introduction

One of the greatest challenges facing modern agriculture is how to enhance soil sustainability without decreasing productivity levels. Among the promising and sustainable proposals for this is the use of natural plant biostimulants (Rouphael and Colla 2018). Plant biostimulants are substances or microorganisms applied to plants to improve the absorption of nutrients, enhance tolerance to abiotic stress, boost crop quality, and maintain soil health (Mannino 2023). These biostimulants are of two main categories: (i) microbial biostimulants, and (ii) non-microbial biostimulants like seaweed extracts, humic substances, protein hydrolysates, chitosan, and other biopolymers (Nephali et al. 2020).

Among biostimulant categories, microbial biostimulants are increasingly favored due to their dynamic interactions with plant roots and their ability to adapt to changing soil conditions. Unlike non-microbial biostimulants, which primarily act through chemical or hormonal pathways, microbial agents can colonize the rhizosphere and continuously contribute to nutrient cycling, stress mitigation, and soil health restoration—especially under challenging environmental conditions (Ali et al. 2024). Plant growth-promoting bacteria (PGPB) are recognized as sustainable biostimulant agents with particular effectiveness under abiotic stress conditions (Kumari et al. 2022). PGPB strains are distinguished based on their plant growth-promoting activities, such as nitrogen fixation, solubilization of nutrients, pathogen suppression, and phytohormone production (Kumari et al. 2022). Microbial consortia have gained growing attention in recent years due to their synergistic interactions and broader functional possibilities compared to single-strain inoculants (Wu et al. 2023). The application of free-living microbial consortia in agricultural soils, however, is generally limited by issues such as low viability, ineffective root colonization, and rapid degradation. Ensuring the viability and functionality of such microbial consortia within the soil ecosystem remains a central challenge (O’Callaghan et al. 2022).

Immobilization of microorganisms on suitable carriers has emerged as a viable solution to these limitations. The selection of carrier material is critical for effective performance, with key criteria including insolubility, biocompatibility, non-toxicity, mechanical and chemical stability, ease of handling, and low cost. Additionally, cell adhesion and porosity are essential, as carriers with high initial-value properties have shown superior performance in microbial immobilization (Mehrotra et al. 2021).

Bacterial cellulose (BC), a biopolymer synthesized by certain bacteria, has been recognized as a good carrier for immobilizing microorganisms due to its high water-holding ability, biocompatibility, and mechanical strength (Gorgieva and Trček 2019). BC is a versatile biomaterial that can be sterilized and processed easily. It has excellent properties, including a highly porous network structure, biocompatibility, and high mechanical and chemical stability. Compared to other carriers such as alginate or synthetic polymers, BC offers distinct advantages: it is fully biodegradable, possesses a highly porous network structure that facilitates nutrient exchange and microbial respiration, and retains moisture exceptionally well (Gorgieva and Trček 2019). Furthermore, BC does not require a lot of treatment to eliminate unwanted polymers or impurities (such as hemicellulose and lignin). It also has high polymerization. BC in its native state possesses a high hydration rate and is able to absorb over a hundred times its weight in water. All these characteristics make BC an excellent immobilization support material for cells (Torres et al. 2012). BC can effectively immobilize microorganisms in a controlled and stable system for release. However, there is relatively limited literature on the effectiveness of BC-immobilized microbial consortia as biostimulants for enhancing plant growth and soil health.

Onion plant (*Allium cepa* L.), a member of the Amaryllidaceae family, is a valuable crop ranked as the fourth most valuable vegetable crop globally (Galdón et al. 2009). Onion bulbs contain vital minerals such as phosphorus and calcium along with protein, vitamin C, quercetin, and flavonoids (Pareek et al. 2017). Onion is the fourth highest export crop in Egypt, behind cotton, rice, and citrus. Onions were selected as the model crop in this study because they are globally economically important and very sensitive to soil nutrient conditions (Ghanem et al. 2021).

The purpose of this study was to investigate the immobilization of a biostimulant microbial consortium on bacterial cellulose (BC) and assess its effect on onion growth, soil nutrient availability, and soil microbial community.

## Materials and methods

### Isolation and identification of cellulose-producing strain

In the current study, a cellulose-producing strain, RY11, was isolated from rotting apples. For the isolation process, rotting apple samples were collected and surface sterilized. Approximately 10 g of rotting apple tissue was collected and homogenized under sterile conditions by crushing. The resultant suspension was filtered through cheesecloth to obtain a liquid suspension, which served as the crude inoculum. Serial dilutions ($\:{10}^{-1}$to $\:{10}^{-7}$) were prepared in sterile saline (0.85% w/v NaCl). The SH (Hestrin-Schramm) medium, containing (w/v): 2% glucose, 0.5% yeast extract, 0.5% peptone, 0.27% $\:{\mathrm{N}\mathrm{a}}_{2}{\mathrm{H}\mathrm{P}\mathrm{O}}_{4}$, and 0.115% citric acid, plus 0.5% (v/v) ethyl alcohol, was prepared and sterilized by autoclaving at 121 °C for 15 min. Erlenmeyer flasks containing 100 mL medium were inoculated with 1% (v/v) of the diluted rotten apple suspension. The inoculated medium was incubated under stationary conditions at 28 °C for 7 d. During incubation, the isolates which formed a visible cellulose pellicle on the medium surface were selected. To obtain pure isolates, the cell suspension was streaked onto SH agar plates (1.5% agar) and incubated at 28 °C for 5 d. Individual colonies were selected (based on morphology), subcultured repeatedly (at least 3 times) to ensure purity, and screened for cellulose production by inoculating into fresh SH liquid medium and confirming pellicle formation after 5 d. Compared to all the cellulose producing strains isolated, RY11 was selected as it exhibited the highest pellicle formation and superior cellulose yield, and hence was taken up for identification and further study.

For identification of selected isolate, genomic DNA was extracted from a 24 h pure culture of strain RY11 using DNA extraction kit (Qiagen, Hilden, Germany) following the manufacturer’s protocol. The strain was identified using 16 S rRNA gene sequencing according to Weisburg et al. (1991). A phylogenetic tree was constructed using the maximum likelihood method with MEGA11 software, and its reliability was tested through bootstrap analysis with 1,000 replications (Tamura et al. 2021). The sequence was deposited in NCBI GenBank and assigned accession number of PX498040.

### Bacterial cellulose production and purification

#### Pre-inoculum preparation and cultivation

To mitigate production costs post-isolation, a pre-inoculum was prepared by subculturing 10% (v/v) of the RY11 culture (from SH medium) into 700 mL of citrus peel hydrolysate medium in a 2 L Erlenmeyer flask. Citrus peels were hydrolyzed following the procedure described by Güzel and Akpınar (2019). Briefly, 10 mL of 0.6 M $\:{\mathrm{H}}_{2}{\mathrm{S}\mathrm{O}}_{4}$ was added to 1 g of dried and ground peels, and the mixture was heated at 100 °C for 2 h to release fermentable sugars like glucose and fructose. After hydrolysis, the mixture was cooled, filtered to remove solids, neutralized to pH 5.5 with NaOH before sterilization. The inoculated medium was incubated at 28 °C under static conditions for 5 d. to promote BC pellicle formation. After cultivation, the bacterial cellulose (BC) samples were purified by treating them with 2% (w/v) NaOH at 80 °C for 90 min to remove bacterial cells and medium components. The samples were subsequently washed with double-distilled water until the pH stabilized (pH 7.0) as described by Żywicka et al. (2016). The BC hydrogel was cut into 1 × 1 $\:{\mathrm{c}\mathrm{m}}^{2}$ pieces and sterilized at 121 °C for 15 min.

#### Bacterial strains and PGP trait assessment

Pure cultures of *Beijerinckia mobilis* (Accession no. PX517308), and *Pseudomonas stutzeri* (Accession no. PX517307) were previously isolated and identified through 16 S rRNA gene sequencing (AbdelRazek and Yaseen 2020). These strains were selected for their complementary plant growth-promoting (PGP) traits. Nitrogen fixation was assessed using the method described by Döbereiner and Day 1976), where the bacterial isolates were grown in nitrogen-free malate medium. Phosphate solubilization was determined following the approach of Liu et al. (2014). HCN production was analyzed by the method of Bakker and Schipper (1987). Indole acetic acid (IAA) estimation was conducted according to the method of Apine and Jadhav (2011). Ammonia production was evaluated based on the procedure described by Dey et al. (2004). Lastly, siderophore production was measured using the method established by Reeves et al. (1983).

#### In vitro compatibility and inoculum preparation

The compatibility of the selected strains was determined in vitro using the cross-streak antagonism method described by Irabor and Mmbaga (2017). One bacterial strain was streaked across a nutrient agar plate, and the other test strain was streaked perpendicular to the first (avoiding contact). Plates were incubated at 28 ± 2 °C and examined for a period of up to 72 h for the presence of a clear zone of inhibition at the intersection, which would indicate antagonism. No zone of inhibition confirmed compatibility.

For preparing broth culture, 1 mL of fresh overnight culture of each bacterium was inoculated into 200 mL of sterilized Nutrient Broth (NB) in a 1 L Erlenmeyer flask (to ensure proper aeration). Subsequently, cultures were incubated at 28 °C with shaking at 150 rpm for 24 h until reaching an OD₆₀₀ of 1.0 (~ 10⁸ CFU/mL).

#### Cell immobilization

Bacterial cells (*B. mobilis* and *P. stutzeri*) were immobilized onto BC using a modified adsorption-incubation protocol adapted from Nguyen et al. (2009). Bacterial cells were harvested from nutrient broth cultures (0.5% w/v peptone, 0.3% w/v beef extract, 0.5% w/v NaCl, pH 7.0) by centrifugation at 8000 rpm at 4 °C for 15 min. The cell pellets were washed twice with sterile 0.9% w/v saline to remove residual medium and resuspended in nutrient broth to a cell density of OD₆₀₀ = 1.0 (~ 10⁸ CFU/mL). The two strains were combined in equal proportions to form a mixed bacterial suspension. Three-day old BC pellicles (1 × 1 $\:{\mathrm{c}\mathrm{m}}^{2}$ pieces, total wet weight) were immersed in the bacterial suspension at a ratio of 20% (w/v), defined as 20 g wet BC per 100 mL suspension. The mixture was incubated at 30 °C on a rotary shaker at 120 rpm for 5 h, conditions optimized for maximum cell adsorption based on preliminary trials. The BC pellicles with immobilized cells were washed three times with sterile Phosphate-buffered saline (PBS; 0.01 M phosphate, 0.137 M NaCl, pH 7.4) to remove unattached cells.

#### Scanning electron microscopy (SEM)

The immobilization efficiency of bacterial cells on the BC matrix was verified using scanning electron microscopy (SEM, Philips XL30). SEM was employed to confirm bacterial attachment and to examine the surface morphology of the BC matrix with immobilized cells. For sample preparation, the BC samples with immobilized cells were fixed in 2.5% glutaraldehyde for 2 h at 4 °C to preserve cellular structures. The samples were then dehydrated using a graded ethanol series (10%, 30%, 50%, 70%, 90%, and 100% ethanol, 15 min per step) to remove water content. Subsequently, the dehydrated samples were sputter-coated with a thin layer of gold (~ 10 nm) to enhance conductivity for SEM imaging. The prepared samples were observed under SEM at an accelerating voltage of 10–20 kV to assess bacterial attachment and the surface morphology of the BC matrix, including fiber structure and bacterial distribution (Cai and Kim 2010).

#### Determination of bacterial survival in bacterial cellulose (BC)

The BC pellicles with immobilized cells were stored at 30 °C for 10, 20, and 30 d to assess the survival of immobilized *B. mobilis* and *P. stutzeri* in BC. For analysis, 1 g of wet BC was added to 9 mL of sterile 0.9% w/v NaCl, vortexed briefly to disrupt the matrix, and incubated at 30 °C on a rotary shaker at 125 rpm for 1 h to release immobilized bacteria (Rezaee et al. 2008). The number of viable bacteria was quantified using the standard plate count technique. Serial dilutions ($\:{10}^{-1}$ to $\:{10}^{-6}$) of the suspension were prepared in 0.9% w/v NaCl, and 100 µL aliquots were spread-plated in triplicate on selective media. *P. stutzeri* was enumerated on King’s B agar containing 2% w/v proteose peptone, 0.15% w/v $\:{\mathrm{K}}_{2}{\mathrm{H}\mathrm{P}\mathrm{O}}_{4}$, 0.15% w/v $\:{\mathrm{M}\mathrm{g}}_{2}{\mathrm{S}\mathrm{O}}_{4}{.7\mathrm{H}}_{2}\mathrm{O}$, 1.5% w/v glycerol, pH 7.2 (King et al. 1954). While *B. mobilis* was quantified on nitrogen-free malate medium containing 0.5% w/v malate, 0.05% w/v $\:{\mathrm{K}}_{2}{\mathrm{H}\mathrm{P}\mathrm{O}}_{4}$, 0.02% w/v $\:{\mathrm{M}\mathrm{g}}_{2}{\mathrm{S}\mathrm{O}}_{4}{.7\mathrm{H}}_{2}\mathrm{O}$, pH 6.8 (Day and Döbereiner 1976). Plates were incubated at 28 °C for 48–72 h, and colony-forming units (CFU/g BC) were counted. Sterile BC without immobilized bacteria served as a negative control to rule out contamination. Three biological replicates were analyzed for each time point.

#### Field experiment

The field experiment was carried out at the Desert Research Center’s experimental research station in El-Kharga Oasis, New Valley, Egypt during the 2024/2025 winter season (November–March). The soil sample was allowed to air dry before being sieved (2 mm/10 mesh) and examined for physical and chemical properties (Page et al. 1982). Soil was sandy clay loam with pH value of 8.15, sodium adsorption ratio of 11.80, electrical conductivity of 7.45 (dS/m), available nitrogen, phosphate, calcium, potassium and magnesium 38.55, 2.35, 32.46, 58.97 and 30.31 mg/kg respectively, organic matter 0.59% and particle size analysis revealed that the soil is composed of 64.44% sand, 7.50% silt, and 28.06% clay. Three soil treatments were applied in this study: (i) soil amended with bacterial cells immobilized in biocellulose (as described above); (ii) soil treated with a liquid bacterial suspension adjusted to a final concentration of $\:{10}^{8}$CFU/kg soil; and (iii) uninoculated control soil.

Bacterial suspensions were prepared by culturing *P. stutzeri* and *B. mobilis* separately in nutrient broth for 24 h at 30 °C and 150 rpm. Each culture was adjusted to a concentration of 10^8^ CFU mL^− 1^ and mixed in a 1:1 ratio prior to application.

A completely randomized design was used, comprising three treatments with three replicates each (*n* = 9 plots). Each experimental plot measured 4 m × 2 m (8 $\:{\mathrm{m}}^{2}$), with 1 m buffer zones between plots to prevent cross-contamination.

Onion (*Allium cepa* L., cv. Giza Red) seedlings (45 days old) were transplanted on both sides of the planting rows at 20 cm spacing within rows and 40 cm between rows.

For the BC-immobilized treatment, 400 g of cell-loaded biocellulose (containing $\:{2.4\:\times\:10}^{8}\:$CFU/g) was evenly distributed and incorporated into each plot. For the liquid treatment, 4 L of bacterial suspension ($\:{10}^{8}$ CFU/kg soil) was applied using a backpack sprayer and raked into the top 10 cm of soil.

Standard local agronomic practices were followed, including application of 150 kg N/ha as urea (split into three doses), 60 kg $\:{\mathrm{P}}_{2}{\mathrm{O}}_{5}$/ha as superphosphate (applied basally), and drip irrigation every 5–7 d.

##### Yield and bulb parameters

Onion bulbs were hand-harvested at optimum bulb maturity (about 150 d after planting). Bulb yield (kg/plot) was recorded at harvesting and later expressed as tons/ha. The weight of ten random bulbs per plot was measured as 10-bulb weight in grams (g). The equatorial diameter of ten bulbs per plot was measured and recorded in centimeters (cm). The freshly harvested bulbs of every plot were weighed at harvesting time as well as after 45 d (Tinna et al. 2020). Physiological loss in weight (PLW) was calculated using the following formula (Tinna et al. 2020):

$$\mathrm{Physiological}\;\mathrm{weight}\;\mathrm{loss}\;(\%)=\frac{\mathrm{Initial}\:\mathrm{wt}-\mathrm{Final}\:\mathrm{wt}}{\mathrm{Initial}\:\mathrm{wt}}\times100$$

### Plant nutrient analysis

Onion bulbs were oven-dried at 60 °C for 72 h until a constant weight was achieved. The dried bulbs were ground and digested following the method described by Bergersen (1980). The concentration of total nitrogen was determined using the micro-Kjeldahl method (Bremner 1960). Total phosphorus content was measured spectrophotometrically through the vanadate molybdate procedure (Koenig and Johnson 1942). Total potassium was quantified using a flame photometer (Banerjee and Prasad 2020). Micronutrient concentrations were analyzed using inductively coupled plasma (ICP) according to the methods outlined by the Association of Official Analytical Chemists (Houba et al. 1996).

### Post-harvest soil sampling and analysis

After harvesting, soil samples were collected at a depth of 0–30 cm from all treatments. The samples were processed and sieved through a 2.0 mm sieve before analysis. Standard protocols (Jackson and Carter 1976) were followed to determine the concentrations of available nitrogen (N), phosphorus (P), potassium (K), and micronutrients.

### Bacterial community structure

Bacterial communities in the soil for different treatments were screened using a high-throughput sequencing technique. DNA was isolated from bulk soil, and six replicates for each of the soil samples were combined to provide homogeneous and sufficient DNA (Ding et al. 2016). DNA was extracted using a QIAamp DNA Stool Mini Kit (Qiagen, Hilden, Germany) following the manufacturer’s instructions for soil samples. The extracted DNA was finally eluted in 50 mL of elution buffer. DNA concentration and purity were initially assessed using a NanoDrop spectrophotometer (Thermo Fisher Scientific, Madison, WI, USA), measuring $\:{\mathrm{A}}_{260/280}$ and $\:{\mathrm{A}}_{260/230}$ ratios to check for protein and humic acid contamination, respectively. Final double-stranded DNA concentration was accurately determined using a Qubit fluorometer to ensure reliability for downstream sequencing applications. DNA integrity was confirmed by agarose gel electrophoresis. The V4–V5 hypervariable region of the bacterial 16 S rRNA gene was amplified using primers 515 F (5′-GTGYCAGCMGCCGCGGTAA-3′) and 926R (5′-CCGYCAATTYMTTTRAGTTT-3′) (Walters et al. 2016). PCR amplification was performed under the following conditions: initial denaturation at 94 °C for 3 min; 35 cycles of 94 °C for 45 s, 55 °C for 60 s, and 72 °C for 90 s; and a final extension at 72 °C for 10 min. Amplicons were purified, indexed, and pooled according to Comeau et al. (2017), then sequenced on the Illumina MiSeq platform (2 × 300 bp paired-end) at the Integrated Microbiome Resource, Dalhousie University, Canada.

### Bioinformatics

Paired-end raw reads were processed using the DADA2 pipeline in R (v3.5.2) (Callahan et al. 2016). The raw fastq files were demultiplexed, quality-checked, filtered, trimmed, and dereplicated. Forward and reverse reads were merged to generate denoised sequences, retaining only those with Phred scores ≥ 30. Chimeric sequences were removed to obtain amplicon sequence variants (ASVs) (Callahan et al. 2016). Taxonomic assignment of ASVs was performed using the *assignTaxonomy* and *assignSpecies* functions against the SILVA v138 reference database. Alpha diversity indices, including Observed ASVs, Chao1, Shannon, and Inverse Simpson, were calculated to evaluate microbial richness and evenness (Callahan et al. 2016). Raw sequence data have been deposited in the NCBI Sequence Read Archive (SRA) under accession number: PRJNA1364181.

### Statistical analysis

All experimental data were subjected to analysis of variance using SPSS (V.19). According to Tukey’s multiple comparison, *P* < 0.05 was regarded as significant in all tests. The comparison of means was made using LSD and Tukey’s HSD.

## Results

### Microbial cellulose production

**Fig. 1:**
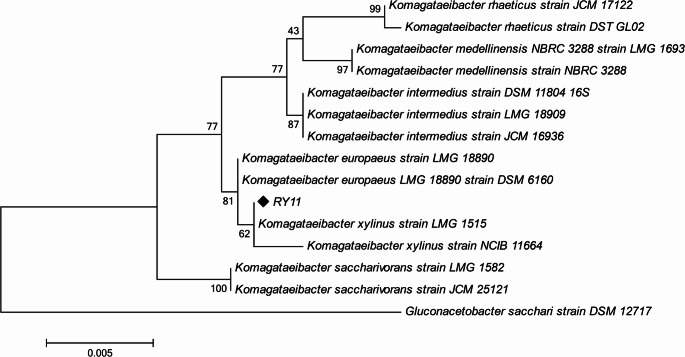
Phylogenetic tree of the isolated biocellulose-producing strain. The tree was constructed using the neighbor-joining method, with bootstrap values (expressed as percentages of 1000 replications) shown at branch points to indicate the reliability of each branch

In this study, the bacterial cellulose producing strain RY11 isolated from a rotten apple was identified as *Komagataeibacter xylinus* (Accession no. PX498113) based on its 16 S rRNA sequencing. The phylogeny was inferred using the Maximum Likelihood method with the Tamura-Nei model (1993) of nucleotide substitutions. The tree with the highest log likelihood (−2,486.06) is shown in Fig. 1. Bootstrap support values (500 replicates) are indicated next to the branches. For the heuristic search, the initial tree was selected based on the superior log-likelihood between a Neighbor-Joining (NJ) tree (Saitou and Nei 1987) and a Maximum Parsimony (MP) tree. The NJ tree was generated from pairwise distances computed using the Tamura-Nei model, while the MP tree represented the shortest length among 10 searches, each starting from a randomly generated tree. The analysis included 15 nucleotide sequences, encompassing $\:{1}^{\mathrm{s}\mathrm{t}}$, $\:{2}^{\mathrm{n}\mathrm{d}}$, $\:{3}^{\mathrm{r}\mathrm{d}}$ codon positions and non-coding regions, with 1,493 positions in the final dataset. Ambiguous positions were removed using the pairwise deletion option. Evolutionary analyses were conducted in MEGA11 (Tamura et al. 2021) with up to three parallel computing threads.

### Plant growth-promoting traits of immobilized strains

The applied bacterial strains (*P. stutzeri* and *B. mobilis*) were tested for their ability to solubilize phosphorus and fix nitrogen (Table 1). Both strains converted insoluble phosphates into plant-available forms, enhancing phosphorus uptake. *P. stutzeri* exhibited higher phosphate solubilization (25.9 µg/mL) than *B. mobilis* (19.2 µg/mL). While *B. mobilis* demonstrated strong nitrogen-fixing capability, *P. stutzeri* did not. Both isolates were also assessed for the production of IAA, siderophores, ammonia, and HCN. Each strain produced IAA, though *P. stutzeri* had slightly higher levels (1.9 µg/mL) compared to *B. mobilis* (1.6 µg/mL). Additionally, *P. stutzeri* produced a significant amount of siderophores (43.4 µg/mL), which chelate iron, improving plant iron uptake while restricting pathogen access. It also produced HCN, whereas *B. mobilis* lacked both siderophore and HCN production. Both strains produced ammonia, contributing to nitrogen availability in the rhizosphere and perhaps antimicrobial activity. The obtained results suggested that combining the two strains could synergistically enhance plant growth promotion. The compatibility of the selected strains was determined in vitro following the method described by Irabor and Mmbaga (2017). No inhibition zones were observed around any of the bacterial colonies, indicating compatibility between strains.

**Table 1:** Multiple PGPR traits of immobilized strains

Isolate	Phosphate Solubilization (µg/mL)	Nitrogen fixation	IAA production (µg/mL)	Siderophore production (µg/mL)	Ammonia Production	HCN production
*P. stutzeri*	25.9	-	1.9	43.4	+	+
*B. mobilis*	19.2	+	1.6	-	+	-

### Viability of immobilized PGPR within bacterial cellulose

The survival of *P. stutzeri* and *B. mobilis* immobilized within bacterial cellulose (BC) was assessed over 30 d of storage at 30 °C. Data presented in Table 2 demonstrated that *B. mobilis* exhibited superior survival ability. It displayed a high cell population of 8.23 × 10^6^ CFU/g after 10 d, which decreased to 5.76 × 10^5^ CFU/g after 20 d and further to 2.45 × 10^5^ CFU/g after 30 d. The nitrogen-fixing capabilities of *B. mobilis* may confer a competitive advantage within the cellulose matrix, potentially providing a source of fixed nitrogen that supports its survival. Also, *P. stutzeri* demonstrated good survival potential within BC. It gave cell counts of 2.19 × 10^6^, 2.64 × 10^5^ and 1.56 × 10^5^ CFU/g after 10, 20 and 30 d respectively.

**Table 2:** Survival of the immobilized PGPR in bacterial cellulose

Isolate	Number of survival cells (CFU/g)		
	10 d	20 d	30 d
*P. stutzeri*	2.19 ± 0.30 × 10^6^	2.64 ± 0.21 × 10^5^	1.56 ± 0.21 × 10^5^
*B. mobilis*	8.23 ± 0.56 × 10^6^	5.76 ± 0.13 × 10^5^	2.45 ± 0.30 × 10^5^

Values are expressed as mean ± Standard Deviation

### Bacterial adhesion on bacterial cellulose surfaces investigated by scanning electron microscopy (SEM)

**Fig. 2:**
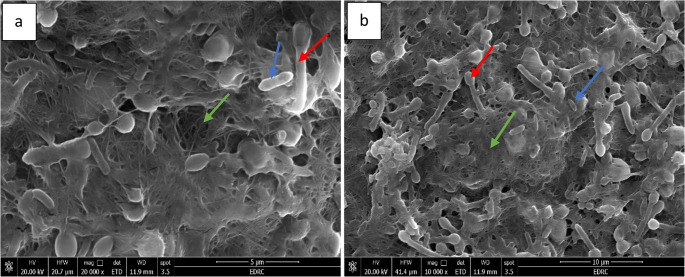
Scanning electron micrograph of immobilized bacterial cells on biocellulose, (**a**) magnification 20,000 and (**b**) magnification 10,000

SEM observations were conducted to evaluate bacterial immobilization on BC. SEM showed that the cellulose nanofibers formed a distinctive multilayer 3D crosslinked weblike porous structure. Figure 2a and b demonstrated that bacteria could establish aggregation structures that allowed them to survive for up to 30 d. Cells of *B. mobilis* (red arrow) are typically rod-shaped with rounded ends and contain unilateral polar lipoid bodies. These bodies are characteristic intracellular inclusions often found at one or both poles of the cells. Meanwhile, cells of *P. stutzeri* (blue arrow) are short rods embedded in cellulose nanofibers, with lower counts compared to *B. mobilis*. The nanoscale fibers are discarded and enmeshed to form a network of thin, porous structure (green arrow).

### Effect of inoculation with immobilized biofertilizer on onion crop yield and physiological loss in bulb weight (PLW) (%)

Results obtained from the study (Table 3) showed that the immobilization treatment significantly increased crop yield when compared with that of free cells inoculation and un-inoculated control. The inoculation with immobilized cells mostly increased crop yield up to 44.90%, while liquid inoculation significantly increased crop yield up to 23.36% when compared with control. The PGPR treatments also, enhanced yield contributing traits (bulb size, fresh and dry weight) compared with un-inoculated control.

**Table 3:** Effect of inoculation with immobilized biofertilizer on onion crop yield and bulb attributes

Treatments	Fresh weight of bulb (g/plant)	Dry weight of bulb (g/plant)	Physiological loss in weight (%)	Equatorial diameter of bulb (cm)	Yield (t/ha)
Control	182.5^b^	93.43^c^	48.5^a^	6.67^c^	20.20^c^
Free cells	227.33^a^	134.17^b^	40.9^b^	7.08^b^	24.92^b^
Immobilized cells	249.17^a^	154.03^a^	37.8^b^	7.84^a^	29.27^a^
LSD at 5%	38.63	13.36	5.11	0.233	2.67

Different letters in the same column indicate significant differences among the treatments according to ANOVA followed by the LSD Fisher’s test (*p* < 0.05). These values are means of three replicates of the treated plants

The maximum physiological loss in weight of bulb % (PLW) (48.5%) was registered with un-inoculated control after 30 d of storage. However, both the immobilized and non-immobilized treatments significantly (*P* < 0.05) reduced physiological loss in weight (PLW) by 22.1% and 15.7%, respectively.

### Plant nutrition and chemical properties of soil

Table 4 showed the nitrogen (N), phosphorus (P), and potassium (K) content in onion bulbs and their availability in the soil at post-harvest. The data revealed that treatments with PGPR enhanced nutrient content in both the bulbs and soil compared to the control. The application of biofertilizer in the form of free cells significantly increased N and P concentrations in the bulbs by 36.5% and 44.4%, respectively, but had no significant effect on K content in the bulbs. Conversely, it significantly boosted the availability of P and K in the soil by 21.5% and 20.8%, respectively, while showing no significant impact on soil-available nitrogen. Bulbs treated with immobilized cells exhibited higher concentrations of N, P, and K, with increases of 49.1%, 58.3%, and 16.5%, respectively, compared to the control. However, there was no significant difference between immobilized and free cells in terms of their effect on N content in the bulbs. Inoculation with immobilized cells also increased the availability of nutrients in the soil, with increases of 17.5 mg/kg, 40.2 mg/kg, and 26.6 mg/kg for available N, P, and K, respectively.

**Table 4:** Effect of inoculation with immobilized biofertilizer on nutrient concentration and post-harvest soil fertility status

Treatments	Bulb nutrient concentration			Soil available NPK		
	*N* (%)	*P* (%)	K (%)	*N* (mg kg^− 1^)	*P* (mg kg^− 1^)	K (mg kg^− 1^)
Control	1.11^b^	0.10^c^	1.96^b^	46.20^b^	5.02^c^	79.4^b^
Free cells	1.75^a^	0.18^b^	2.08^b^	49.65^b^	6.4^b^	100.33^a^
Immobilized cells	2.18^a^	0.24^a^	2.35^a^	56.00^a^	8.4^a^	108.20^a^
LSD at 5%	0.56	0.03	0.2712	4.49	0.94	11.08

Different letters in the same column indicate significant differences among the treatments according to ANOVA followed by the LSD Fisher’s test (*p* < 0.05). These values are means of three replicates of the treated plants

### Trace element content in soil and onion bulb

The data in Table 5 demonstrated that both free and immobilized bacterial cell treatments significantly enhanced micronutrient content in onion bulbs and soil compared to the control. In onion bulbs, immobilized cells led to the highest concentrations of calcium (16.64 mg/kg), copper (0.06 mg/kg), iron (1.79 mg/kg), magnesium (9.66 mg/kg), manganese (1.09 mg/kg), and zinc (0.43 mg/kg), with statistically significant differences (*p* ≤ 0.05). Similarly, in soil, immobilized cells markedly increased trace element levels, especially iron (3.88 mg/kg), manganese (3.79 mg/kg), and calcium (77.75 mg/kg), surpassing both the control and free cell treatments.

**Table 5:** Effect of inoculation with immobilized biofertilizer on micronutrients content in onion bulb and rhizospheric soil

Treatments	Micronutrient content in onion bulb					
	Ca	Cu	Fe	Mg	Mn	Zn
Control	12.30^c^	0.04^b^	1.37^b^	7.53^c^	0.05^a^	0.20^b^
Free cells	15.05^b^	0.06a^b^	2.77^b^	8.45^b^	0.09^a^	0.31a^b^
Immobilized cells	16.64^a^	0.10^a^	7.74^a^	9.66^a^	1.09^a^	0.43^a^
LSD at 5%	0.77	0.0.05	1.53	0.69	1.84	0.13
Micronutrient content in soil						
Control	58.50^b^	0.11^a^	1.60^c^	33.55^b^	2.56^b^	0.57^b^
Free cells	74.60^a^	0.28^a^	2.90^b^	34.28^b^	3.27^ab^	0.63^b^
Immobilized cells	77.57^a^	0.42^a^	4.86^a^	38.23^a^	3.79^a^	0.76^a^
LSD at 5%	4.03	0.34	0.211	1.49	1.01	0.10

Different letters in the same column indicate significant differences among the treatments according to ANOVA followed by the LSD Fisher’s test (*p* < 0.05). These values are means of three replicates of the treated plants

### Determination of NPK uptake in bulb

The application of PGPR treatments significantly increased the uptake of nitrogen, phosphorus, and potassium by onion bulbs compared to the control. Inoculation with bacterial free cells enhanced N, P, and K uptake by 48.5%, 54.9%, and 23.6%, respectively. However, immobilized cells demonstrated a more pronounced effect, with N, P, and K uptake increasing by 64.8%, 71.2%, and 42.4%, respectively (Table 6).

**Table 6:** Effect of inoculation with immobilized biofertilizer on total nutrient uptake by onion

Treatments	*N* uptake (Kg ha^− 1^)	*P* uptake (Kg ha^− 1^)	K uptake (Kg ha^− 1^)
Control	22.4^c^	2.02^c^	39.59^c^
Free cells	43.6^b^	4.5^b^	51.83^b^
Immobilized cells	63.8^a^	7.02^a^	68.78^a^
LSD at 5%	11.7	1.9956	6.4916

Different letters in the same column indicate significant differences among the treatments according to ANOVA followed by the LSD Fisher’s test (*p* < 0.05). These values are means of three replicates of the treated plants

### Soil bacterial community composition

**Fig. 3:**
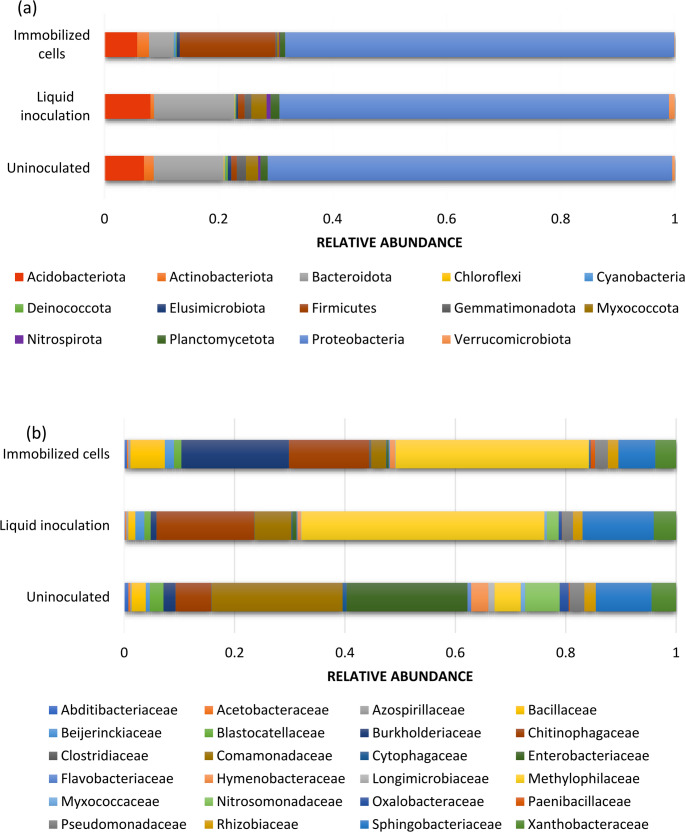
Taxonomic composition of the onion rhizosphere microbiome. Relative abundance (%) is shown at (a) the phylum level and (b) the family level. Stacked bar charts illustrate the microbial community structure under different treatments

The impact of inoculation strategies on soil bacterial communities was investigated by examining their composition and diversity. Abundant classes observed in this study in general, were Proteobacteria, Bacteroidota, Acidobacteriota, Firmicutes, Actinobacteriota, Myxococcota, Gemmatimonadota and Planctomycetota. Data in Fig. (3a) revealed distinct shifts in soil bacterial phyla between treatments. Proteobacteria remained dominant in the onion rhizosphere across all samples (68–70%). However, the specific inoculation strategies induced notable alterations in other phyla. The immobilized cell treatment led to a distinct increase in Firmicutes (16.20%) and Actinobacteria (2.00%) compared to both free cells and the control (1.2% and 0.97% Firmicutes and 0.61% and 1.7% for Actinobacteria respectively). Conversely, Bacteroidetes showed a significant decrease in the immobilized cell treatment (4.10%) compared to free cells (13.46%) and control (12.12%). The relative abundance of Acidobacteriota phylum increased with liquid inoculation (7.68%) beyond immobilized treatment (5.54%) and control (6.85%).

At family level, the data presented in Figure (3b) revealed distinct microbial community dynamics across treatments. The uninoculated control was dominated by Comamonadaceae (4.39%) and Enterobacteriaceae (4.06%). Methylophilaceae increased dramatically in liquid inoculation (21.6%) and immobilized cells (20.8%) compared to uninoculated (0.88%), suggesting a strong response to inoculation. Burkholderiaceae shows a massive increase in immobilized cells (11.59%) compared to uninoculated (0.41%) and liquid inoculation (0.52%). Both Beijerinckiaceae and Pseudomonadaceae populations in the onion rhizosphere significantly increased with inoculation. Liquid inoculation led to increases of 0.8% and 0.98% for each, respectively, while immobilized cells resulted in higher increases of 0.97% and 1.38% respectively. While the uninoculated control showed only 0.13% and 0.5%. Chitinophagaceae is highly abundant in both inoculation methods (8.6–8.7%) compared to uninoculated (1.19%). Comamonadaceae decreases from 4.39% (uninoculated) to 3.27% (liquid) and further to 1.6% (immobilized). Enterobacteriaceae drops sharply from 4.06% (uninoculated) to 0.36% (liquid) and 0.22% (immobilized). Immobilized cells favor Burkholderiaceae, Methylophilaceae, and Chitinophagaceae, with higher Paenibacillaceae (1.38%) compared to liquid (0.97%).

### Ternary diagram of soil microbial community under different inoculation strategies

**Fig. 4:**
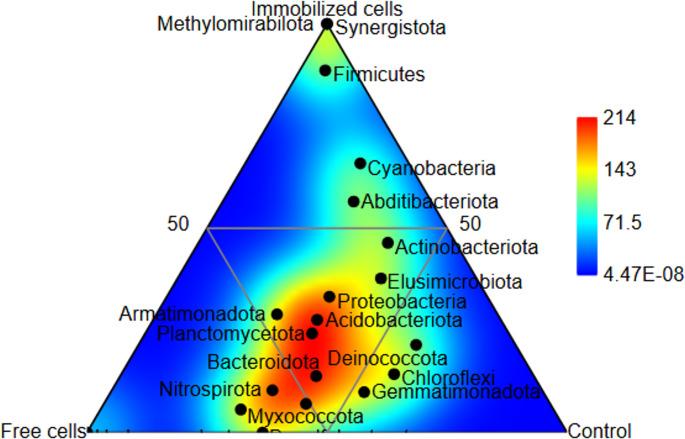
Ternary visualization of soil microbial community

The ternary plot provides a complementary view, and used to compare the distribution of bacterial phyla in the onion rhizosphere across different inoculation strategies (Fig. 4). Each corner represents 100% influence from each condition. The position of each phylum within the triangle indicates its distribution. The color gradient represents the abundance distribution of the community. The red/yellow regions of the plot represent high-levels of the relative abundance, while the blue regions represent low-levels of the relative abundance. Proteobacteria dominates all conditions, occupying a central position, suggesting it is a core rhizosphere component regardless of treatment. Firmicutes clusters near the immobilized cells corner, highlighting a strong preference for this strategy, while Bacteroidota and Myxococcota are more associated with free cells and control. Rare phyla like Cyanobacteria and Fusobacteriota appear only in immobilized cells, implying niche-specific enrichment. The plot reveals that immobilized cells favor distinct taxa (e.g., Firmicutes, Actinobacteriota), free cells promote others (e.g., Bacteroidota), and the control maintains a more balanced but unique composition, demonstrating that inoculation methods significantly reshape microbial community structure.

#### Bacterial diversity and richness indices

Alpha diversity analysis provided descriptive insights into the bacterial community richness, diversity, and evenness across the three soil treatments. Alpha diversity was assessed using several metrics: Chao1, Shannon diversity index, Inverse Simpson index, and dominance. As shown in Table 7, the un-inoculated soil displayed the highest observed richness, recording a Chao1 index of 324. Immobilization appeared to reduce species richness by approximately 30% compared to the control, and similar trends were observed for the Shannon and Inverse Simpson indices. This observation suggests that both Plant Growth-Promoting Rhizobacteria (PGPR) treatments led to a reduction in bacterial richness and diversity within the analyzed pooled sample. The highest dominance was observed in the formulated cells treatment (0.3005), which may suggest that the formulated strains successfully proliferated and became the most prevalent groups in that pooled soil sample. Conversely, the un-inoculated soil exhibited the lowest dominance (0.2773), which is consistent with a more even distribution of bacterial species. These findings indicate a tendency for the PGPR-inoculations, especially those with strains immobilized in biocellulose, to reduce the overall observed soil bacterial diversity and richness. This reduction is interpreted as the introduced strains effectively proliferating and becoming the dominant bacterial groups.

**Table 7:** Ecological indexes calculated from Illumina sequencing results

Treatments	Alpha diversity				
	Individuals	Chao1	Shannon	InvSimpson	Dominance
Control	100,004	324	2.482	1.38	0.2773
Free cells	107,636	262	2.405	1.41	0.2889
Immobilized cells	129,152	226	2.177	1.43	0.3005

## Discussion

In this study, the bacterial cellulose-producing *K. xylinus* strain RY11 was used as a microbial factory for BC production. This BC was used as a carrier of natural origin, as it is characterized by different porosity-related parameters, including specific surface area, pore size, and pore volume, as well as various values of mechanical strength (Żywicka et al. 2019). Two PGP bacterial strains (*P. stutzeri* and *B. mobilis*) were selected based on their compatibility. The BC was soaked in the cell suspension, and cells were adsorbed mostly on the surface of the BC due to its porous nanostructure. Nguyen et al. (2009) reported that adsorption increased significantly with BC surface area, and the highest adsorption efficiency was achieved for the “youngest” 3-day-old BC pellicles, which displayed the largest surface area. The larger the surface area, the more space is available between BC fibrils so that more cells can adsorb onto and penetrate into the carrier. Similarly, the size and shape of the immobilized cells also affect adsorption efficiency.

The survival of *P. stutzeri* and *B. mobilis* immobilized within bacterial cellulose (BC) was assessed over 30 d of storage at 30 °C. Both strains maintained high viability and showed strong survival within the BC matrix. The high metabolic diversity of immobilized strains likely enables them to utilize components of the bacterial cellulose matrix as a nutrient source, thereby supporting prolonged survival (Rius et al. 2001). Consistent with these observations, Khongkool et al. (2025) reported that BC provides a protective environment for immobilized microorganisms, not only sustaining their survival but also potentially enhancing their functional performance.

SEM analysis was conducted to evaluate bacterial immobilization on BC. Cells of *B. mobilis* appeared rod-shaped with rounded ends and contained polar lipoid bodies, primarily composed of poly-β-hydroxybutyrate, which function as energy reserves (Dedysh et al. 2005). In contrast, *P. stutzeri* cells were observed as short rods, present in lower abundance, and embedded within cellulose nanofibers. These nanofibers formed a thin, porous network that facilitated bacterial diffusion and uniform distribution throughout the sponge-like structure and along fiber surfaces (Santaolalla et al. 2021).

BC serves as an excellent immobilization matrix for plant growth-promoting rhizobacteria (PGPR) due to its distinctive nanostructure and environmentally compatible properties (Gorgieva and Trček 2019). Unlike synthetic polymers or alginate, BC exhibits very high porosity (> 90%) with pore sizes ranging from 50 to 500 nm, forming a three-dimensional nanofibrillar network. This architecture supports high cell loading (> 10⁹ CFU/g wet BC), efficient nutrient and gas exchange, and sustained release of microbial metabolites into the rhizosphere (Ashrafi et al. 2019). Moreover, BC is fully biodegradable, being degraded by soil cellulases within 60–90 d, thereby leaving no carrier residues and minimizing ecological impact, unlike non-degradable clays or slow-degrading biochar. Importantly, BC can be synthesized in situ in the presence of PGPR, enabling co-entrapment during pellicle formation. This approach preserves cell viability and metabolic activity more effectively than post-immobilization encapsulation (Khan and Kamal 2021).

The distinctive properties of BC make it an effective carrier for PGPR, particularly in onion cultivation. Onion is a shallow-rooted, nutrient-demanding crop that often struggles with nutrient acquisition, as essential nutrients frequently leach beyond the reach of its root system (Arunachalam et al. 2024). This limitation highlights the need for sustained and localized microbial activity in the rhizosphere to ensure continuous micronutrient solubilization and growth promotion. To address this challenge, plant growth-promoting rhizobacteria (PGPR) were immobilized within bacterial cellulose (BC), creating a stable and localized microbial presence around onion roots. To validate this approach, a field trial was conducted during the 2025 winter cropping season at El-Kharga Oasis, New Valley Governorate, Egypt. Results showed that PGPR treatments, especially in their immobilized form, significantly improved onion yield and its contributing traits compared with the uninoculated control. Although numerous greenhouse studies have reported growth promotion with immobilized PGPR, evidence from field-scale trials under arid conditions remains limited. The enhanced productivity observed in inoculated plants was likely due to plant growth-promoting substances secreted by rhizobacteria (Ajijah et al. 2023), which strengthen root systems and enhance nutrient and water uptake, thereby overcoming the limitations typically faced by onion crops. Similar improvements in onion yield and nutrient uptake under arid conditions were reported by Arunachalam et al. (2024) and Blanco et al. (2023), who demonstrated the effectiveness of phosphorus-solubilizing bacteria and native PGPR strains, respectively. Field trials in Egypt further confirmed that biofertilizer inoculation combined with mineral nitrogen enhanced onion productivity and storability under arid climates (Hamdtah et al. 2025).

Previous studies have reported that survival and colonization of PGPR inoculants can be improved through bacterial encapsulation, which enhances plant performance and soil fertility under competitive soil environments, including drought and saline conditions (Szopa et al. 2022; Zhang et al. 2023). In addition, both immobilized and non-immobilized PGPR treatments significantly (*P* < 0.05) reduced physiological loss in bulb weight. This effect may be attributed to increased dry matter and total dissolved solids in bulbs. Onions with higher dry matter content are firmer and possess thicker, better-adhering skins, enabling them to retain water more effectively than bulbs with lower dry matter, higher water content, and thinner skins (Kiran et al. 2024).

The data further indicate that PGPR treatments enhanced nutrient content, both macro- and micronutrients, in onion bulbs and surrounding soil relative to the uninoculated control. Variations in NPK levels across different treatments may be influenced by several factors, including weathering processes, upward movement of soluble ions through capillary action, and decomposition of plant residues (Prasad 2022). Inoculation with PGPR addresses two key objectives of modern agriculture: increasing crop yield and improving the nutritional quality of seeds and fruits (Kumari et al. 2023). Numerous studies have confirmed the beneficial effects of PGPR on onion, demonstrating their ability to enhance both productivity and nutritional composition (Petrovic et al. 2020; Pellegrini et al. 2021; Arunachalam et al. 2024). Findings show that PGPR application, especially via immobilized cells, markedly boosts micronutrient accessibility in the rhizosphere and enhances bulb nutrient absorption. Elevations in elements like Fe, Mg, and Zn within bulbs and soil can likely be attributed to the extended release and persistent function of immobilized PGPR, which optimize nutrient solubilization and root performance (Riseh et al. 2021). These findings highlight the potential of immobilized PGPR as a sustainable biofertilizer to improve soil health, enhance nutrient acquisition, and increase yields, particularly under micronutrient-deficient conditions. The results align closely with reports from Arunachalam et al. (2024), who noted substantial improvements in onion growth, nutrient efficiency, and output following inoculation with phosphorus-solubilizing bacteria. Overall, the findings suggest that immobilization enhances the effectiveness of PGPR in promoting nutrient uptake. Bacterial inoculation, especially in immobilized form, improves nutrient bioavailability and uptake, thereby contributing to soil fertility and crop nutritional quality. PGPR act as biofertilizers by producing growth regulators such as auxins and gibberellins, which stimulate root development and expansion, increasing the plant’s capacity to absorb nutrients (Abdelhameid 2020). Moreover, PGPR inoculation likely promotes root elongation in onion, enhancing nutrient acquisition, stress resilience, and systemic resistance (Gill et al. 2016). This combined effect can enlarge root surface area, facilitating superior absorption of macronutrients (N, P, K, S) (Li et al. 2023) and micronutrients (Padash et al. 2016).

The impact of inoculation strategies on soil bacterial communities was assessed by analyzing their composition and diversity. Across all treatments, Proteobacteria remained the dominant phylum in the onion rhizosphere, consistent with previous reports of their ecological importance in crop soils (Spain et al. 2009; Miyashita 2015; Kim et al. 2021). In contrast, immobilized cell treatments induced notable shifts in other phyla, with marked increases in Firmicutes and Actinobacteria. The enrichment of Firmicutes, known for their role in organic matter decomposition and plant growth promotion, suggests that the bacterial cellulose (BC) matrix provides a favorable microenvironment. Similarly, the rise in Actinobacteria, often linked to soil health and antibiotic production, indicates a beneficial restructuring of the microbial community. These results align with Wang et al. (2019), who observed similar increases in rice rhizospheres following biofertilizer application. Conversely, Bacteroidetes declined under immobilized treatments, likely due to competitive exclusion by faster-growing taxa, consistent with findings from Xie et al. (2025) and Pongsilp and Nimnoi (2024).

At the family level, distinct dynamics were observed. The uninoculated control was dominated by Comamonadaceae and Enterobacteriaceae, while inoculation, particularly immobilized treatments, enhanced Methylophilaceae, Burkholderiaceae, Bacillaceae, Beijerinckiaceae, and Pseudomonadaceae. The suppression of Enterobacteriaceae in PGPR treatments parallels biocontrol mechanisms where beneficial taxa competitively exclude opportunistic pathogens (Köberl et al. 2017). Moreover, PGPR consortia enriched functional families such as Sphingomonadaceae and Xanthobacteraceae (pollutant degradation), Oxalobacteraceae (heavy metal immobilization), and Nitrospiraceae (nutrient cycling), underscoring their potential for both agricultural productivity and bioremediation (Liu et al. 2022; Rani et al. 2023).

Alpha diversity analysis suggested differences in bacterial community richness, diversity, and evenness among the three soil treatments. Uninoculated soils exhibited the highest richness (Chao1 index = 324), whereas immobilization reduced species richness and evenness. While these findings point to reduced bacterial diversity and richness following PGPR inoculation, particularly with strains immobilized in biocellulose, they should be interpreted cautiously, as results are based on a single replicate without statistical confirmation. However, this pattern reflects selective enrichment of introduced strains, consistent with Malusà et al. (2016) and Pongsilp and Nimnoi (2024) who noted that immobilized inoculants often decrease richness while enhancing functional diversity. Importantly, the BC matrix, a natural hydrogel, protected *P. stutzeri* and *B. mobilis*, maintaining their viability and metabolic activity throughout cultivation. This persistence directly influenced soil community composition and functional efficiency. PGPR inoculants reshape soil bacterial communities through multiple mechanisms, including nutrient solubilization (phosphorus, iron, ammonia), modulation of stress responses, phytohormone production (e.g., IAA), and exudate release that alters microbial interactions. The observed reduction in richness after inoculation likely reflects the selective effects of root exudates favoring beneficial taxa (Kong and Liu 2022; Tang et al. 2023).

While many greenhouse studies have reported growth promotion with immobilized PGPR, evidence from field-scale trials under arid conditions remains scarce. The present findings therefore provide important validation that BC-immobilized PGPR can overcome the nutrient limitations typically faced by onion crops, offering a promising strategy for sustainable yield improvement under stress-prone environments.

## Conclusion

The present investigation confirms that the immobilization of PGPR (*Pseudomonas stutzeri* and *Beijerinckia mobilis*) within a biocellulose matrix represents a superior and sustainable biostimulant strategy. Inoculation significantly (𝑃<0.05) enhanced onion plant growth in both free and immobilized forms compared to the control, with the immobilized formulation consistently outperforming free cells. Notably, immobilized PGPR increased bulb yield by 42%, improved soil fertility, and enhanced nutrient mobilization, leading to higher N, P, and K uptake in onion bulbs. Ecological diversity indices (Chao1, Shannon, and Inverse Simpson) revealed a short-term reduction in microbial richness and diversity, interpreted as evidence of targeted microbiome engineering where, introduced PGPR selectively suppressed non-beneficial competitors, thereby improving rhizosphere efficiency.

While these findings validate microbial immobilization as a robust, eco-friendly agricultural tool, the study is limited by its short-term field trial and focus on a single crop and soil type beside pooled replicates for DNA sequencing, leaving just one sample per soil type. This reduced the ability to confirm differences in microbial communities. To strengthen the approach, future research should include long-term trials across different seasons, soils, and crops, with individual replicates analyzed separately. Such studies will be critical to fully establish microbial immobilization as a cornerstone of sustainable agriculture.

## Data Availability

No datasets were generated or analysed during the current study.

## Abbreviations

BC: Bacterial cellulose
SH: Hestrin-Schramm
PGPR: Plant growth-promoting rhizobacteria
IAA: Indole acetic acid
CFU: Colony-forming units
SEM: Scanning Electron Microscopy
NB: Nutrient Broth
